# Vitamin D Insufficiency Exacerbates Adipose Tissue Macrophage Infiltration and Decreases AMPK/SIRT1 Activity in Obese Rats

**DOI:** 10.3390/nu9040338

**Published:** 2017-03-29

**Authors:** Eugene Chang, Yangha Kim

**Affiliations:** Department of Nutritional Science and Food Management, Ewha Womans University, Seoul 03760, Korea; eugenics77@hotmail.com

**Keywords:** adenosine monophosphate-activated protein kinase (AMPK), adipose tissue macrophage infiltration, obesity, sirtulin 1 (SIRT1), vitamin D

## Abstract

Obesity is recognized as a state of chronic low-grade systemic inflammation due to adipose tissue macrophage infiltration and production of proinflammatory adipokines. Decreased vitamin D status is associated with obesity. The specific aim of the present study is to investigate the effects of vitamin D on obesity-induced adipose tissue inflammation. Male Sprague-Dawley rats were randomized and fed a normal diet (NOR, 1000 IU vitamin D/kg diet), a 45% high-fat diet (HF, 1000 IU vitamin D/kg diet), or a 45% high-fat diet containing 25 IU vitamin D/kg diet (HF+LVD) for 12 weeks. The vitamin D-insufficient diet (HF+LVD) led to vitamin D inadequacy as determined by serum 25(OH)D level, 68.56 ± 7.97 nmol/L. The HF+LVD group exacerbated HF-increased adipocyte size, adipogenic gene expression of PPARγ, adipose tissue macrophage recruitment, and proinflammatory cytokine IL-6 and TNFα levels in epididymal white adipose tissue. In addition, vitamin D insufficiency significantly decreased mRNA levels of β-oxidation-related genes such as CPT1α, PGC1α, PPARα, VLCAD, LCAD, MCAD, and UCP1. Moreover, significant decrements of SIRT1 and AMPK activity were noted in obese rats fed with a vitamin D-insufficient diet. The observed deleterious effects of vitamin D insufficiency on adipose tissue expansion, immune cell infiltration and inflammatory status suggest vitamin D plays a beneficial role in adipocyte metabolic metabolism and obesity progression. SIRT1 and AMPK activity may play a role in the mechanism of vitamin D action.

## 1. Introduction

Obesity is characterized by excessive fat accumulation in adipose tissue [1,2]. Increased adipose tissue mass is associated with changes in the endocrine and metabolic functions of adipose tissue, reflecting the increased number of infiltrated immune cells [3,4,5] and the production and secretion of biologically active proteins, including leptin, tumor necrosis factor α (TNFα), interleukin-6 (IL-6), monocyte chemoattractant protein-1 (MCP-1), resistin and adiponectin [6,7]. Increased adiposity and local inflammation in adipose tissue are linked to alterations in systemic physiology and the pathogenesis of obesity-induced complications [8,9,10]. Thus, an understanding of the molecular mechanisms of adipose tissue formation, function and changes during the progression of obesity is required for the prevention and treatment of obesity and obesity-related complications.

There is a strong association between vitamin D status and obesity, as low vitamin D status is highly prevalent in obese people [11,12]. Negative relationships between body fat content and 25-hydroxyvitamin D (25(OH)D), the most accepted marker of vitamin D status, have been reported [13,14,15,16]. In parallel with body fat mass, the synthesis and release of proinflammatory adipocyte-derived proteins are also affected by vitamin D status. Vitamin D deficiency, also known as hypovitaminosis D, is positively associated with serum levels of inflammatory markers, such as IL-6, TNFα and C-reactive protein in obese participants [17,18]. In this respect, the influence of vitamin D on inflammation has been investigated using in vitro adipocytes. In some in vitro studies, vitamin D shows proinflammatory properties [19,20], whereas anti-inflammatory pathways are activated with similar vitamin D concentrations in adipocytes [21,22,23,24]. Recently published studies show that gene modification of the vitamin D receptor (VDR) regulates fatty acid oxidation, energy metabolism and browning of white adipose tissue in part through UCP1 expression [25,26], demonstrating regulation of vitamin D in obesity. In addition, 1,25-dihydroxyvitamin D3 treatment suppresses brown adipocyte differentiation and mitochondrial respiration [27]. Because of discrepancies between the observed effects of vitamin D on inflammation and the role of vitamin D in regulating brown adipose tissue development and function, the mechanisms by which vitamin D influences adipocyte tissue formation, adipokine production and secretion and adipose tissue inflammation during the development of obesity need to be elucidated by further investigation. 

Adenosine monophosphate-activated protein kinase (AMPK) and sirtuin 1 (SIRT1), a nicotinamide adenine dinucleotide (NAD)-dependent protein deacetylase, have emerged as critical energy sensors and inflammatory regulators. In the visceral adipose tissue of obese humans, reduced AMPK activity is closely associated with adipose tissue inflammation [28]. Studies in genetically obese and high-fat-diet-fed rodents demonstrate that body fat deposition is influenced by AMPK activation [29,30,31]. AMPK enhances SIRT1 by increasing NAD/NADH ratio and decreases adipose tissue macrophage infiltration and inflammation [32,33,34]. In addition, AMPK and SIRT1 act as regulators of fatty acid oxidation and mitochondrial biogenesis by phosphorylation and deacetylation of peroxisome proliferative activated receptor gamma coactivator 1α (PGC1α) [35]. SIRT1 protects diet-induced obesity and inflammation and obesity-associated metabolic dysfunction [36,37,38,39]. In addition, vitamin D decreases adipocyte fat accumulation and increases SIRT1 activation in adipocytes [40]. Thus, AMPK and SIRT1 activation have been proposed as key regulators to prevent obesity and obesity-related metabolic dysfunction. In the present study, we investigate the effects of vitamin D on fat accumulation, adipose tissue macrophage infiltration, proinflammatory cytokines and AMPK/SIRT1 activity in diet-induced obese rats.

## 2. Materials and Methods

### 2.1. Animals and Diets

Animal housing and procedures were approved by the Ethics Committee for Animal Experiments of the Ewha Womans University in Seoul, South Korea (permission number: 2015-15-064). Three-week-old male Sprague-Dawley rats (*n* = 23) were obtained from Doo Yeol Biotech (Seoul, Korea), housed individually in wire-mesh-bottomed polypropylene cages and maintained in a UVB light-free environment on a 12-h light/dark cycle at constant room temperature (22 ± 2 °C) and humidity (55% ± 5%). After 1 week of acclimation with laboratory chow, the rats were randomly divided into three diet groups (*n* = 7–9/group, Table 1): (1) normal diet (NOR, 10% fat diet with 1000 IU vitamin D/kg diet); (2) high-fat diet (HF, 45% fat diet with 1000 IU vitamin D/kg diet); or (3) 45% fat diet containing 25 IU vitamin D/kg diet (HF+LVD). In the present study, dietary vitamin D levels were chosen to achieve target 25(OH)D status levels of inadequacy and adequacy without adverse effects [41,42,43]. All diets were prepared by Research Diets, Inc. (New Brunswick, NJ, USA). Body weight and food intake were monitored twice a week. All rats and food were weighed every Monday and Thursday between 09:00 and 11:30. Food was weighed before it was given to each rat in each cage. To measure food intake, both split food and remaining uneaten food were weighed and subtracted from weighed food before being provided to the animals. At the end of the 12-week animal experiment, the rats were fasted overnight and killed by CO_2_ narcosis. After blood collection by cardiac puncture, the samples were centrifuged at 1500× *g* for 20 min at 4 °C to obtain serum and stored at −20 °C until analysis. Serum measurements of 25(OH)D, metabolic parameters, cytokines and SIRT1 activity were conducted. Tissues were dissected, weighed, frozen in liquid nitrogen and stored at −70 °C for further analysis, or were fixed, embedded in paraffin and sectioned for histologic analysis. 

### 2.2. Measurements of Serum Metabolic Parameters

Serum concentrations of alanine transaminase (ALT), aspartate transaminase (AST), glucose, total cholesterol (TC), triglycerides (TG), high-density lipoprotein (HDL) cholesterol and low-density lipoprotein (LDL) cholesterol were measured using an automatic enzymatic procedure (Roche Diagnostics, Mannheim, Germany) and a Hitachi 7600 autoanalyzer (Tokyo, Japan), according to the manufacturers’ recommended protocols. Body vitamin D status was evaluated using a rat 25(OH)D enzyme-linked immunosorbent assay (ELISA) kit (ALPCO, Salem, NH, USA). In the first step, 25(OH)D was bound to a specific monoclonal antibody. A fixed amount of biotinylated 25(OH)D was conjugated with tetramethylbenzidine (TMB) chromogenic substrate at room temperature in the presence of horseradish peroxidase (HRP). Exposure to direct sunlight was avoided. The amount of substrate turnover was determined calorimetrically by measuring the absorbance at 450 nm, which is inversely proportional to the total 25(OH)D (D2 and D3) concentration. Measurement of serum insulin was carried out using a radioimmunoassay (RIA) method with a rat insulin RIA kit (Linco Research, Inc., St. Charles, MO, USA). ^125^I-labeled insulin and a rat insulin antiserum were utilized to determine the level of rat insulin in serum separated with appropriate dosages of anticoagulant by the double antibody/PEG technique. 

### 2.3. Histological Analysis of Adipose Tissue and Cell Size Determination

Epididymal adipose tissue (EAT) was harvested, fixed in 4% formaldehyde overnight, embedded in paraffin, sectioned and stained with hematoxylin and eosin (H&E). Digital images were acquired with a microscope (Olympus, Tokyo, Japan). The size of the adipocyte area was determined using Image J software (National Institutes of Health, Bethesda, MD, USA).

### 2.4. Immunohistochemistry and Crown-Like Structure (CLS) Quantification

Histological sections of adipose tissue were dewaxed in xylenes, rehydrated and stained using monoclonal anti-mouse F4/80 antibody (Serotec, Raleigh, NC, USA). A Polink-2 Plus HRP Anti-rat DAB Detection kit was used (Golden Bridge International, Inc., Mukilteo, WA, USA) according to the manufacturer’s instructions. Subsequently, the reaction products were visualized using diaminobenzidine (Golden Bridge International, Inc.) and counterstained with Mayer hematoxylin (ScyTek, Logan, UT, USA). Adipocyte death was quantified by the number of crown-like structures (CLS) within histologic sections of epididymal adipose tissue in which F4/80-positive macrophages surround a single adipocyte.

### 2.5. RNA Isolation, Reverse Transcription and Real-Time Quantitative Polymerase Chain Reaction (RT-PCR)

Isolation of RNA from epididymal adipose tissue was performed using an RNeasy Lipid Tissue Mini Kit (QIAGEN, Valencia, CA, USA) according to the manufacturer’s protocol. cDNA was synthesized from 1 μg of total RNA using a MMLV Reverse Transcriptase Kit (Bioneer, Daejeon, Korea). The reaction was performed at 37 °C for 60 min followed by incubation at 95 °C for 5 min. Primers used are shown in Table 2. The polymerase chain reaction (PCR) parameters were as follows: pre-denaturation at 95 °C for 10 min, followed by 50 cycles of denaturation at 95 °C for 15 s, annealing at 60 °C for 20 s and extension at 72 °C for 20 s. Data were analyzed using the ∆∆Ct method for relative quantification [44]. Expression of each target was normalized to the average of β-actin as a control and expressed as the fold change related to the NOR group.

### 2.6. Cytokine Assay

Concentrations of IL-6 and TNFα in serum and adipose tissue were measured using Quantikine ELISA kits (R&D systems, Minneapolis, MN, USA) according to the manufacturer’s protocols. Quantitative sandwich enzyme immunoassay technique utilized specific monoclonoal antibodies for rat IL-6 and TNFα under light protection. Color intensity in proportion to the amount of IL-6 and TNFα was measured at 450 nm. Epididymal adipose tissue was homogenized in radioimmunoprecipitation assay (RIPA) buffer (Sigma-Aldrich, St. Louis, MO, USA) with 1% protease inhibitor cocktails (Roche, Mannheim, Germany). Cytokine levels in adipose tissue were normalized to their respective protein concentrations and expressed as ng/mg protein.

### 2.7. SIRT1 Activity Assay

SIRT1 activity was measured using an SIRT1 activity assay kit (Abcam, Cambridge, MA, USA). After extracting the nuclear fraction, fluorescence intensity was detected at 340 nm excitation and 460 nm emission using a microplate fluorescence reader (Varioskan Flash, Thermo Scientific, Waltham, MA, USA). Protein was determined using a BCA protein assay kit (Thermo Scientific). SIRT1 activity was normalized to their respective protein concentrations and expressed as the fold change compared to the NOR group.

### 2.8. AMPK Activity Assay

AMPK activity was measured using an AMPK Kinase Assay kit (MBL International Co., Woburn, MA, USA). AMPK activity was semi-quantified using mouse IRS-1 serine 789, which can be phosphorylated by AMPK. Colorimetric reaction was developed by peroxidase conjugated anti-mouse IgG and TMB. The absorbance was measured at 450 nm using a microplate reader. AMPK activity was normalized to their respective protein concentrations and expressed as the fold change compared to the NOR group.

### 2.9. Statistical Analysis

Data are presented as the mean ± standard error of the mean (SEM). Statistical differences between two and three groups were determined by Student’s *t*-test or one-way analysis of variance (ANOVA) following Student-Newman-Keuls multiple comparisons method. Statistical significance was defined at *p* < 0.05 using SPSS software (version 21; IBM Corporation, Armonk, NY, USA).

## 3. Results

### 3.1. Dietary Vitamin D-Insufficient Diet Exacerbates High Fat Diet-Increased Body Weight Gain and Fat Deposition

**Figure 1:**
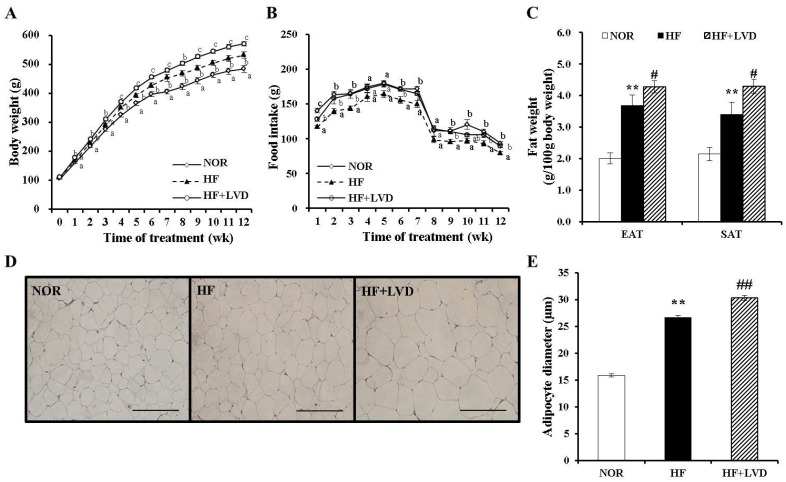
Effects of vitamin D-insufficient diet on body weight gain and fat weight in obese rats. Body weight gain (**A**), food intake (**B**) and weight of epididymal adipose tissue (EAT) and subcutaneous adipose tissue (SAT) (**C**). Representative hematoxylin and eosin stained EAT section (scale bar, 50 μm; magnification, 40×) (**D**). Average adipocyte diameter of adipocytes within adipose tissue (**E**) and data is expressed as the mean ± SEM. Bars with different letters (a, b, c) show significant difference (*p* < 0.05). * *p* < 0.05; ** *p* < 0.01 compared to NOR. # *p* < 0.05; ## *p* < 0.01 compared to HF. NOR, 10% fat diet with 1000 IU vitamin D (*n* = 9); HF, 45% fat diet with 1000 IU vitamin D (*n* = 7); HF+LVD, 45% fat diet containing 25 IU vitamin D (*n* = 7).

A total of 23 Sprague-Dawley rats were fed 10% fat or 45% fat diets containing 25 or 1000 IU vitamin D/kg diet for 12 weeks. There was no significant difference in initial body weight, energy efficiency, or weights of liver or skeletal muscle (Figure 1A, Table 3). The vitamin D-insufficient diet (HF+LVD) group showed a significant increase in body weight compared to the vitamin D adequate diet (HF) group, despite equal food efficiency and energy efficiency (Figure 1A, Table 3). The HF+LVD group displayed significant increase in adipose tissue weight (Figure 1C) and adipocyte size (Figure 1D,E) compared to the HF group. These results demonstrate that vitamin D insufficiency exacerbates diet-induced obesity and adipose tissue expansion.

### 3.2. Influence of a Vitamin D-Insufficient Diet on Serum Vitamin D and Metabolic Parameters

Next, we investigated the effect of dietary vitamin D levels on blood 25(OH)D concentration, the best blood indicator of vitamin D status. Regardless of fat content in the diet, the NRC vitamin D level requirement, 1000 IU vitamin D/kg diet [41], led to a 25(OH)D concentration over 100 nmol/L, which was proposed to be optimal. In contrast, serum 25(OH)D levels in 25 IU vitamin D/kg diet (HF+LVD) was less than 80 nmol/L, defined as vitamin D inadequacy or insufficiency in humans [45,46]. This demonstrates that dietary vitamin D levels affect the systemic vitamin D status in the body. 

During the progression of obesity, the influence of vitamin D insufficiency on changes in blood metabolic characteristics was determined using enzymatic assays using commercial kits. Among HF-increased serum levels of insulin, TG, TC, LDL-cholesterol, serum insulin, TC and LDL-cholesterol concentrations were exacerbated by vitamin D insufficiency. Serum levels of AST and ALT were not statistically different among the groups (Table 4).

### 3.3. Influene of Vitamin D Insufficiency on Gene Expression invovled in Adipogensis and Fat Oxidation

**Figure 2:**
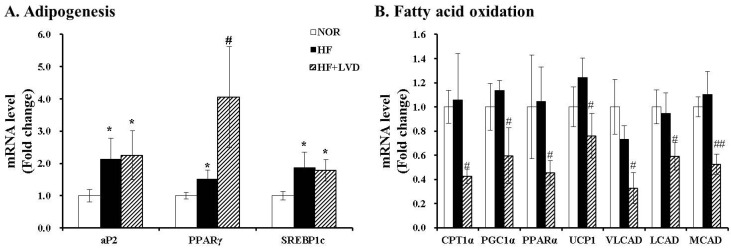
Influence of vitamin D insufficiency on gene expression involved in adipogenesis (**A**) and fatty acid oxidation (**B**). mRNA levels were determined by RT-PCR and normalized for all samples to β-actin. The results are expressed as the fold change compared to NOR. The value of each bar represents the mean ± SEM. * *p* < 0.05; ** *p* < 0.01 compared to NOR. # *p* < 0.05; ## *p* < 0.01 compared to HF. NOR, 10% fat diet with 1000 IU vitamin D (*n* = 9); HF, 45% fat diet with 1000 IU vitamin D (*n* = 7); HF+LVD, 45% fat diet containing 25 IU vitamin D (*n* = 7).

We evaluated whether vitamin D inadequacy exacerbates HF-induced body weight gain and fat deposition by gene expression related to adipogenesis or fatty acid oxidation in adipose tissue. mRNA levels of adipogenic genes, such as fatty acid-binding protein 2 (aP2), peroxisome proliferator activated receptor γ (PPARγ) and sterol regulatory element binding protein 1c (SREBP1c), were measured using RT-PCR. Adipogenic gene levels of aP2, PPARγ and SREBP1c were significantly increased by the HF diet (*p* < 0.05). The vitamin D-insufficient diet (HF+LVD) led to a significant induction of only PPARγ gene expression, approximately 2.7-fold higher than that of the HF diet (Figure 2A). In contrast, mRNA levels involved in β-oxidation, such as carnitine palmitoyltransferase 1α (CPT1α), PGC1α, PPARα, very long-chain acyl-CoA dehydrogenase (VLCAD), long-chain acyl-CoA dehydrogenase (LCAD) and medium-chain acyl-CoA dehydrogenase (MCAD), as well as gene expression of uncoupling protein 1 (UCP1) related to thermogenesis and energy expenditure, were significantly suppressed by vitamin D inadequacy (Figure 2B).

### 3.4. Vitamin D-Insufficient Diet Increases Inflammatory Cytokines in Serum and Adipose Tissues of Obese Rats

**Figure 3:**
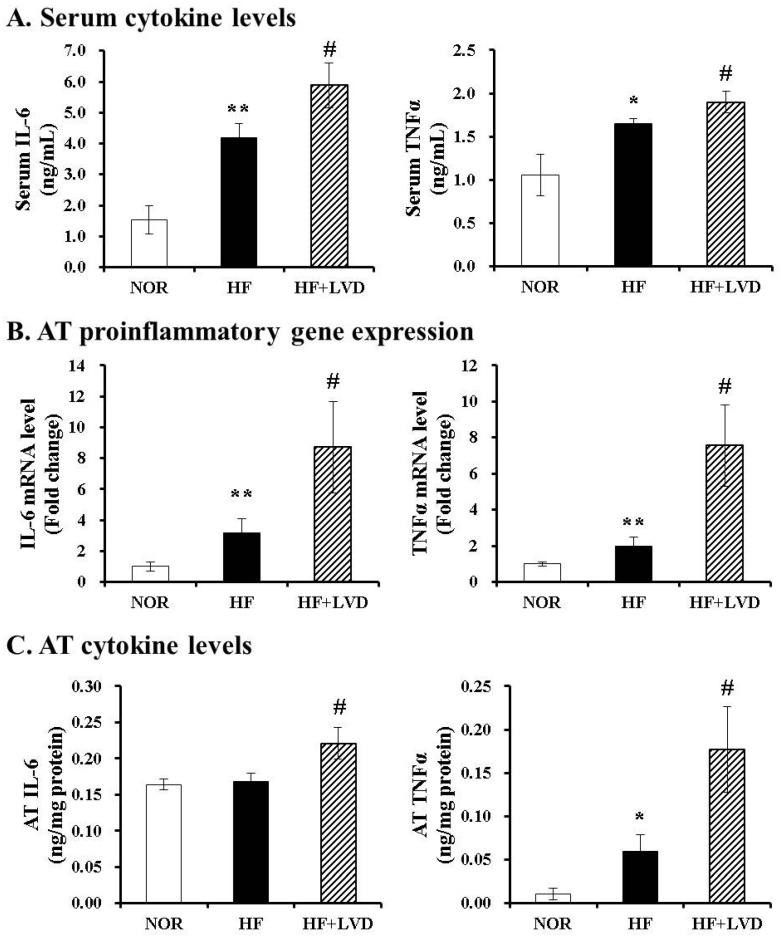
Vitamin D insufficiency increases local and systemic proinflammatory cytokine level. Serum levels of IL-6 and TNFα were expressed as ng/mL (**A**); Gene expression was measured, normalized for all samples to β-actin and expressed as the fold change compared to NOR (**B**); Measurements of IL-6 and TNFα in adipose tissue (**C**) were normalized to their respective protein concentrations and expressed as ng/mg protein. Values are expressed as the mean ± SEM. * *p* < 0.05; ** *p* < 0.01 compared to NOR. # *p* < 0.05 compared to HF. NOR, 10% fat diet with 1000 IU vitamin D (*n* = 9); HF, 45% fat diet with 1000 IU vitamin D (*n* = 7); HF+LVD, 45% fat diet containing 25 IU vitamin D (*n* = 7).

To demonstrate the effect of vitamin D inadequacy on local and systemic inflammation, the production and secretion of proinflammatory cytokines in serum and adipose tissue were evaluated using commercial colorimetric enzyme-linked kits and RT-PCR. Vitamin D insufficiency (HF+LVD) significantly exacerbated HF-increased serum concentrations of IL-6 and TNFα by 2.73- and 1.56-fold, respectively (Figure 3A). Consistent with serum levels, there were significant 2.75- and 3.82-fold increase in IL-6 and TNFα mRNA levels between vitamin D-insufficient and optimal levels in the high-fat diet group (Figure 3B). In addition, vitamin D insufficiency led to increased levels of IL-6 and TNFα in adipose tissue by 1.31- and 2.98-fold, respectively (Figure 3C). This indicates that vitamin D inadequacy might result in increased local and systemic inflammation during the development of obesity.

### 3.5. Vitamin D Insufficiency Significantly Increases Macrophage Infiltration in Obese Adipose Tissue

**Figure 4:**
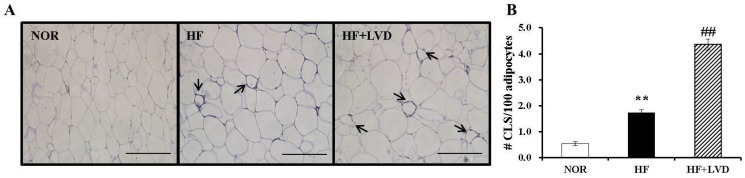
Vitamin D insufficiency increases macrophage infiltration in adipose tissue. (**A**) Macrophage marker, F4/80 immunohistochemistry of epididymal adipose tissue (scale bar, 50 μm; magnification, 40×); the black arrows indicate a CLS; (**B**) Number of CLS was quantified from multiple histologic sections and expressed as the mean ± SEM. ** *p* < 0.01 compared to NOR. ## *p* < 0.01 compared to HF. NOR, 10% fat diet with 1000 IU vitamin D (*n* = 9); HF, 45% fat diet with 1000 IU vitamin D (*n* = 7); HF+LVD, 45% fat diet containing 25 IU vitamin D (*n* = 7).

To investigate the effect of a vitamin D-insufficient diet on obesity-associated adipose tissue inflammation, F4/80 immunohistochemistry was carried out on epididymal adipose tissue samples. In the present study, HF significantly increased F4/80 localization around adipocytes and the number of crown-like structures (CLS) by 3.22-fold compared to NOR (*p* < 0.05). The HF+LVD group showed a significant 2.51-fold increase in CLS formation compared to the HF group (Figure 4A,B). Thus, vitamin D insufficiency might lead to adipose tissue macrophage recruitment during the development of obesity.

### 3.6. Vitamin D Insufficiency Decreases AMPK and SIRT1 Activity in Obese Adipose Tissue

**Figure 5:**
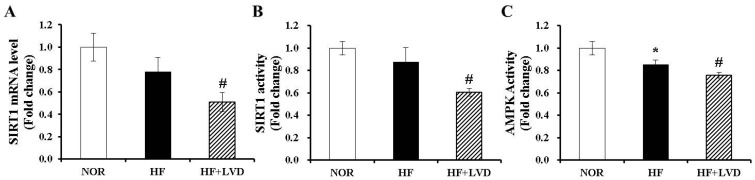
Vitamin D insufficiency decreases mRNA expression and activity of SIRT1 and AMPK activity in adipose tissue. SIRT1 mRNA levels were measured by quantitative RT-PCR and normalized to β-actin (**A**); SIRT1 activity was analyzed by a fluorometric SIRT1 activity assay kit (**B**); AMPK activity was measured using an AMPK kinase kit, normalized to their relative protein contents and expressed as the fold change compared to NOR (**C**). The value of each bar represents the mean ± SEM. * *p* < 0.05 compared to NOR. # *p* < 0.05 compared to HF. NOR, 10% fat diet with 1000 IU vitamin D (*n* = 9); HF, 45% fat diet with 1000 IU vitamin D (*n* = 7); HF+LVD, 45% fat diet containing 25 IU vitamin D (*n* = 7).

The activities of two important nutrient sensors and inflammatory regulators, AMPK and SIRT1, in adipose tissue were determined in the current study. Vitamin D insufficiency (HF+LVD) significantly reduced both SIRT1 gene expression and activity compared to the HF group (Figure 5A,B). Next, we examined whether vitamin D inadequacy affects AMPK activity in addition to decreasing SIRT1 activity. As shown in Figure 5C, there was a further inhibitory effect of vitamin D insufficiency on adipose tissue AMPK activation (*p* < 0.05).

## 4. Discussion

Accumulating evidence demonstrates a negative association between vitamin D status and obesity [11,12]. The present study determined that diet-induced vitamin D insufficiency exacerbated high-fat-diet-increased body weight gain, adipose tissue expansion and macrophage infiltration and inflammation. To the best of our knowledge, this study demonstrates for the first time that vitamin D insufficiency significantly decreases SIRT1 and AMPK activity in the adipose tissues of obese rats. These results suggest that increased vitamin D intake might have beneficial effects on obesity due to reduced fat accumulation and decreased local and systemic inflammation, concurrent with an increase in AMPK/SIRT1 activity. 

During the progression of obesity, adipose tissue exhibits dynamic expansion, increased production and secretion of peptides and inflammation [1,3,6,7,9]. Targeting adipose development and function changes during the development of obesity has been regarded as a possible strategy for the prevention and/or treatment of obesity and its-associated metabolic disorders. Low vitamin D status is prevalent among the obese, and low vitamin D status has been linked with an increased risk of adiposity [11,13,14]. Increased dietary vitamin D intake is related to lower visceral adiposity and adipocyte size [47]. Therefore, we first examined the influence of dietary vitamin D levels on HF-increased body weight and adipose tissue expansion. In the present study, a 1000 IU vitamin D/kg diet—the NRC requirement for rodent vitamin D level [41]—yielded optimal 25(OHD) serum levels, a commonly accepted indicator of vitamin D status. By contrast, the 25 IU vitamin D/kg diet used in the present study led to vitamin D insufficiency in humans [45,46]. However, it is possible that a high-fat diet per se reflects blood 25(OH)D levels. Therefore, a follow-up study including a NOR+LVD group might be needed. Vitamin D insufficiency exacerbated HF-induced body weight, consistent with previous in vivo animal studies showing the protective effects of vitamin D on diet-induced obesity [48,49]. Moreover, vitamin D inadequacy (HF+LVD) significantly increased adipocyte size and adipogenic gene expression of PPARγ, a transcription factor regulating adipocyte differentiation and lipogenesis in epididymal adipose tissue, without changing food efficiency or energy efficiency, compared to the 45% fat diet. Discrepant food intake according to vitamin D levels in the diet and a sharp decline in food intake starting in 8 week of study were not fully resolved. Further research is warranted to explore the effects of vitamin D insufficiency on food intake and decrements in food consumption. 

Fatty acid oxidation occurs in cooperation with CPT1α-mediated entry of long-chain fatty acids into mitochondria [50] and a transcription factor, PGC1α. PGC1α promotes fatty acid oxidation by mitochondrial biogenesis and oxidative metabolism in association with its nuclear receptor, PPARα [51]. Moreover, acyl-CoA dehydrogenases (ACADs), such as VLCAD, LCAD and MCAD catalyze the rate-limiting step in the mitochondrial β-oxidation [52,53]. The HF+LVD group displayed significantly decreased mRNA levels of CPT1a, PGC1α, PPARα, VLCAD, LCAD and MCAD. These data suggest that vitamin D insufficiency-increased adipose tissue expansion might be associated with decreased fatty acid oxidation capacity. However, it is not clear whether decreased mRNA levels related to β oxidation are the direct results of vitamin D insufficiency or an indirect consequence of diet-induced obesity. To investigate the precise mechanisms by which vitamin D inadequacy exacerbates diet-induced obesity, further studies with NOR+LVD group need to directly measure fatty acid oxidation, oxygen consumption, mitochondrial respiration and physical activity. In addition, vitamin D insufficiency significantly reduced mRNA expression of UCP1, which is involved in thermogenesis, energy expenditure and the browning of white adipose tissue [54,55]. Adipose specific vitamin D receptor (VDR) overexpression decreases energy expenditure and fatty acid oxidation in conjunction with reduced UCP1 gene expression in brown adipose tissue [26]. However, the possibility that vitamin D promotes browning of white adipose tissue in the progression of diet-induced obesity remains to be determined in further studies. 

A positive association between low vitamin D status and inflammation has been found in obese subjects [17,18]. Vitamin D supplementation in high-fat diet decreases IL-6 production in murine adipose tissue [56]. In LPS-injected and diet-induced obese mice, 4-day gavage administration of cholecalciferol—a native form of vitamin D—inhibits epididymal adipose tissue inflammation and macrophage infiltration [57]. In addition to adipose tissue, hepatic inflammatory and oxidative stress genes were upregulated by a vitamin D-depleted diet [58]. Consistent with these previous studies, the results from the present study also demonstrated that vitamin D insufficiency significantly increased proinflammatory gene expression of IL-6 and TNFα in adipose tissue and concentrations of IL-6 and TNFα in both adipose tissue and serum. In addition, adipose tissue macrophage infiltration, as demonstrated by numbers of CLS (F4/80-positive adipocyte surrounded by macrophages), was exacerbated by vitamin D inadequacy. These results indicate that vitamin D inhibits diet-induced adiposity/obesity and obesity-associated inflammation. However, vitamin D insufficiency-increased adipose tissue inflammation might be the consequence of diet-induced adipose expansion/obesity together with increased inflammation in other tissues such as liver, subcutaneous adipose tissue, or brown adipose tissue. Therefore, further studies are warranted to investigate the precise contribution of vitamin D insufficiency to local and systematic inflammation by including a NOR+LVD group and measuring inflammation markers in not only epididymal adipose tissue but also other tissues. 

Accumulating evidence suggests that two important nutrient sensors and inflammatory regulators, AMPK and SIRT1, act as pathogenic factors for adipocyte formation and adipose tissue inflammation and macrophage infiltration during the development of obesity. Close links between reduced AMPK activity, adiposity and inflammation have been reported in the adipose tissue of obese patients and genetically or diet-induced obese rodents [28,29,31]. Moreover, mice lacking an AMPKα subunit show increased adiposity and adipocyte hypertrophy [59]. Chronic chemical activation of AMPK decreases lipogenesis and triglyceride synthesis and inhibits the expression and secretion of proinflammatory cytokines [60,61]. These results demonstrate that AMPK activation has adipocyte metabolic functions, which have been linked to protection against obesity and obesity-associated inflammation. In the present study, we revealed that a decrement of AMPK activation and increments of gene expression and concentrations of IL-6 and TNFα in the adipose tissues of HF-fed obese rats were exacerbated by vitamin D insufficiency. In addition, we found that diet-induced vitamin D insufficiency significantly decreased both SIRT1 expression and activity, consistent with our previous in vitro study. In murine adipocytes, vitamin D treatment remarkably decreased adipocyte lipid storage and increased SIRT1 expression and activation [37]. Numerous studies demonstrate an inverse relationship between SIRT1 and adipose tissue mass and inflammation [37,38,39]. SIRT1 is regulated by AMPK activation in energy homeostasis and metabolism [32,33,34]. Moreover, AMPK and SIRT1 promote lipid catabolism by direct phosphorylation and interaction with PGC1α [35]. Given the close relationship between AMPK and SIRT1 activation and their effects on obesity and its-associated inflammation, AMPK/SIRT1 activity could be a target for prevention or treatment of obesity-associated endocrine and metabolic effects. Taken together, our data suggest that vitamin D levels in the diet influence adipose tissue mass and function in a way that may positively modulate AMPK/SIRT1 activity. Despite important findings illustrating vitamin D insufficiency in diet-induced obesity and obesity-associated inflammation, our study has limitations stemming from gender bias. Like previous other studies, the present study used only male animals to prevent confounding factors such as reproductive cycles and hormone fluctuations. To ascertain exactly how vitamin D affects adipose tissue formation and function, especially in women, and to translate our findings to the clinical practice, further studies with female animal models are necessary. 

## 5. Conclusions

The current study demonstrates that vitamin D insufficiency exacerbates high-fat-diet-increased adipose tissue expansion and macrophage recruitment, as well as the expression and secretion of proinflammatory adipokines. Vitamin D insufficiency significantly increases adipogenic gene expression and decreases mRNA levels involved in fatty acid oxidation and AMPK/SIRT1 activity in epididymal adipose tissue. Thus, our findings suggest that increased dietary intake of vitamin D might be a possible strategy for obesity prevention and treatment. In addition, our findings raise the new possibility that vitamin D-mediated AMPK/SIRT1 activity could be a target for obesity prevention or treatment.

## Figures and Tables

**Table 1:** Content of intervention diets.

	NOR	HF	HF+LVD
Casein (g)	200	200	200
l-Cystine (g)	3	3	3
Corn starch (g)	550	72.8	72.8
Maltodextrin (g)	150	100	100
Sucrose (g)	0	172.8	172.8
Cellulose (g)	50	50	50
Soybean oil (g)	25	25	25
Lard (g)	20	177.5	177.5
Total kcal	4057	4057	4057
Carbohydrate (% of energy)	70	35	35
Fat (% of energy)	10	45	45
Vitamin D (IU/kg diet)	1000	1000	25

NOR, 10% fat diet with 1000 IU vitamin D; HF, 45% fat diet with 1000 IU vitamin D; HF+LVD, high-fat plus low vitamin D, 45% fat diet containing 25 IU vitamin D.

**Table 2:** Primers used for Real-Time Quantitative Polymerase Chain Reaction (RT-PCR).

Gene	GeneBank No.	Forward Sequence (5′-3′)	Reverse Sequence (5′-3′)	Product Size (bp)
aP2	NM_053365	TCACCCCAGATGACAGGAAA	CATGACACATTCCACCACCA	140
β-actin	NM_031144	GTCGTACCACTGGCATTGTG	TCTCAGCTGTGGTGGTGAAG	180
CPT1α	NM_031559.2	ATGACGGCTATGGTGTCTCC	GTGAGGCCAAACAAGGTGAT	154
IL-6	NM_012585	ATAGTCCTTCCTACCCCAAC	TGCCGAGTAGACCTCATAGT	143
LCAD	NM_012819.1	CCTACAGCTGCATGAAACCA	GACGATCTGTCTTGCGATCA	229
MCAD	NM_016986.2	TATGCCCTGGACAGGAAAAC	CCTTCGCAATAGAGGCAAAG	172
MCP-1	NM_031530	ACTCACCTGCTGCTACTCAT	CTACAGCTTCTTTGGGACAC	101
PGC1α	NM_031347.1	ATGAGAAGCGGGAGTCTGAA	TGCATTCCTCAATTTCACCA	159
PPARα	NM_013196.1	TACCTGTGAACACGATCTGA	GCTAGTCTTTCCTGCGAGTA	136
PPARγ	NM_001145366	TGTGGGGATAAAGCATCAGC	CAAGGCACTTCTGAAACCGA	175
SIRT1	XM_008774951.1	AGGGAACCTCTGCCTCATCT	GAGGTGTTGGTGGCAACTCT	199
SREBP1c	AF286470	AGGAGGCCATCTTGTTGCTT	GTTTTGACCCTTAGGGCAGC	134
TNFα	NM_012675	CCCCTTTATCGTCTACTCCT	ACTACTTCAGCGTCTCGTGT	139
UCP1	NM_012682.2	GACTCGGATCCTGGAACGTC	GCATAGGAGCCCAGCATAGG	151
VLCAD	NM_012891.2	GCATCTTGCTCTATGGCACA	ACTTTCCACAGGGGCTAGGT	156

aP2, fatty acid-binding protein 2; CPT1α, carnitine palmitoyltransferase 1α; IL-6, interleukin-6; LCAD, long-chain acyl-CoA dehydrogenase (Acadl); MCAD, medium-chain acyl-CoA dehydrogenase (Acadm); MCP-1, monocyte chemoattractant protein-1; PGC1α, peroxisome proliferative activated receptor gamma coactivator 1α; PPARα, peroxisome proliferator-activated receptor α; PPARγ, peroxisome proliferator-activated receptor γ; SIRT1, sirtuin 1; SREBP1c, sterol regulatory element-binding protein1c; TNFα, tumor necrosis factor α; UCP1, uncoupling protein 1; VLCAD, very long-chain acyl-CoA dehydrogenase (Acadvl).

**Table 3:** Effects of vitamin D on body weight, food intake and tissue weight.

	NOR (*n* = 9)	HF (*n* = 7)	HF+LVD (*n* = 7)
Body weight (g)			
- Initial body weight	105.86 ± 2.64	109.69 ± 2.50	110.75 ± 3.36
- Final body weight	483.84 ± 11.49 ^a^	532.21 ± 10.36 ^b^	571.08 ± 7.52 ^c^
- Weight change	377.98 ± 11.63 ^a^	422.53 ± 10.65 ^b^	458.03 ± 8.91 ^c^
Food intake (g/day)	20.66 ± 0.37 ^b^	18.03 ± 0.43 ^a^	20.03 ± 0.37 ^b^
Energy intake (kcal/day)	79.43 ± 1.42 ^a^	85.23 ± 2.02 ^b^	94.68 ± 1.75 ^c^
Food efficiency (g gain/g consumed)	0.22 ± 0.005 ^a^	0.28 ± 0.004 ^b^	0.28 ± 0.004 ^b^
Energy efficiency (g gain/kcal consumed)	0.057 ± 0.002	0.060 ± 0.000	0.059 ± 0.001
Tissue weight (g/100 g body weight)			
- Liver	2.87 ± 0.06	3.05 ± 0.11	3.14 ± 0.11
- Skeletal muscle	0.68 ± 0.03	0.61 ± 0.04	0.66 ± 0.05

Data are expressed as the mean ± SEM (*n* = 7–9/group). Significant differences among groups are represented with different letters (a, b, c) at *p* < 0.05. NOR, 10% fat diet with 1000 IU vitamin D; HF, 45% fat diet with 1000 IU vitamin D; HF+LVD, high-fat plus low vitamin D, 45% fat diet containing 25 IU vitamin D.

**Table 4:** Metabolic serum characteristics of low-fat control (NOR) and high fat control (HF) and vitamin D-insufficient (HF+LVD)-fed rats.

	NOR (*n* = 9)	HF (*n* = 7)	HF+LVD (*n* = 7)
25(OH)D (nmol/L)	102.59 ± 6.75	103.46 ± 5.76	68.56 ± 7.97 ^#^
Glucose (mmol/L)	12.28 ± 0.98	14.03 ± 2.40	16.74 ± 1.95 *
Insulin (μU/mL)	39.90 ± 4.19	58.77± 5.06 **	75.56 ± 8.17 ^#^
Lipids (mmol/L)			
- Triglyceride	0.87 ± 0.10	1.22 ± 0.08 *	1.80 ± 0.28 *
- Total cholesterol	1.97 ± 0.13	2.63 ± 0.13 **	3.56 ± 0.54 ^#^
- HDL cholesterol	2.13 ± 0.10	2.35 ± 0.10	2.35 ± 0.21
- LDL cholesterol	0.21 ± 0.03	0.29 ± 0.03 **	0.39 ± 0.03 ^##^
AST (IU/L)	107.50 ± 11.82	107.00 ± 13.85	107.83 ± 12.97
ALT (IU/L)	33.63 ± 6.45	30.60 ± 4.23	30.71 ± 2.56

Data are expressed as the mean ± SEM (*n* = 7–9/group). * *p* < 0.05; ** *p* < 0.01 compared to NOR. # *p* < 0.05; ## *p* < 0.01 compared to HF. NOR, 10% fat diet with 1000 IU vitamin D; HF, 45% fat diet with 1000 IU vitamin D; HF+LVD, high-fat plus low vitamin D, 45% fat diet containing 25 IU vitamin D. 25(OH)D, 25-hydroxyvitamin D; HDL, high-density lipoprotein; LDL, low-density lipoprotein; AST, aspartate aminotransferase; ALT, alanine aminotransferase.
